# Microbial processes with the potential to mobilize As from a circumneutral-pH mixture of flotation and roaster tailings

**DOI:** 10.1038/s41598-023-50435-3

**Published:** 2023-12-27

**Authors:** Eva Pakostova, David M. Hilger, David W. Blowes, Carol J. Ptacek

**Affiliations:** 1 Department of Earth and Environmental Sciences, University of Waterloo, Waterloo, Canada; 2 Centre for Manufacturing and Materials, Coventry University, Coventry, UK

**Keywords:** Element cycles, Element cycles, Environmental monitoring

## Abstract

The Northwest Tailings Containment Area at the inactive Giant Mine (Canada) contains a complex mixture of arsenic-containing substances, including flotation tailings (84.8 wt%; with 0.4 wt% residual S), roaster calcine wastes (14.4 wt% Fe oxides), and arsenic trioxide (0.8 wt%) derived from an electrostatic precipitator as well as As-containing water (21.3 ± 4.1 mg L^−1^ As) derived from the underground mine workings. In the vadose zone the tailings pore water has a pH of 7.6 and contains elevated metal(loid)s (2.37 ± 5.90 mg L^−1^ As); mineral oxidizers account for 2.5% of total 16S rRNA reads in solid samples. In the underlying saturated tailings, dissolved Fe and As concentrations increase with depth (up to 72 and 20 mg L^−1^, respectively), and the mean relative abundance of Fe(III)-reducers is 0.54% of total reads. The potential for As mobilization via both reductive and oxidative (bio)processes should be considered in Giant Mine remediation activities. The current remediation plan includes installation of an engineered cover that incorporates a geosynthetic barrier layer.

## Introduction

The Giant Mine (Yellowknife, NT, Canada) produced 220 t of Au from 1948 to 2004^1^. Most Au in the Giant deposit is hosted within arsenopyrite (FeAsS) and As-rich pyrite (FeS_2_). A roaster complex operated from 1949 to 1999 to oxidize the sulfide concentrate and produce an iron oxide calcine to achieve Au recovery via cyanidation^2^. The initial roasting activities released > 20,000 t of As_2_O_3_ through stack emissions^3^, resulting in high As levels in local soils and waters and both acute and chronic exposure to As^4^. Emission control measures, including installation of an electrostatic precipitator (ESP), were implemented (in 1958) and a tailings effluent treatment system was constructed (in 1981)^5^. The ESP produced an As_2_O_3_-rich dust—arsenic trioxide roaster waste (ATRW)—that was collected and stored in mined-out stopes and purpose-built vaults and chambers at the mine site. Currently, 237,000 t of ATRW is stored underground at the Giant Mine. The release of dissolved As from the ATRW, as well as from As-contaminated water pumped from underground workings, is an ongoing environmental concern.

As-sulfide bearing flotation tailings (16 million t), mixed with roaster calcine wastes containing on average 3000 mg kg^−1^ As and stored in above-ground tailings impoundments, represent another major source of potential environmental risk at the Giant Mine. In addition, water from the underground mine workings, containing high dissolved As (21.7 ± 3.4 mg L^−1^; 2016–2022), was pumped into and retained in a collection pond in the Northwest Tailings Containment Area (NW-TCA). A remediation strategy for the tailings impoundments is being developed with the main objective of preventing the mobilization of As. The remediation plan includes drainage of the facilities, regrading to improve drainage, and installation of a non-vegetated engineered cover that incorporates a geosynthetic barrier layer^6^.

Oxidative dissolution of sulfidic minerals in the vadose zone is commonly a major cause of As mobilization from sulfide-rich mine wastes. Total S content of the Giant Mine tailings is low (0.39 wt%; mainly as arsenopyrite and arsenical pyrite)^5^. Neutral pH mine environments, such as the Giant Mine tailings, support the growth of neutrophilic S-oxidizing microorganisms (SOM; e.g., *Thiobacillus thioparus*) and Fe(II)-oxidizing microorganisms (IOM; e.g., *Gallionella* and *Sideroxydans*). These chemolithotrophic prokaryotes have been detected in circumneutral mine wastes in significant numbers^7–9^, while acidophilic species (e.g., *Acidithiobacillus*) can be present in lower numbers^8^.

Roasting sulfide concentrates during ore processing oxidizes Fe and drives off S as SO_2_, resulting in the formation of roaster calcine composed of oxidized Fe-bearing phases (e.g., hematite [α-Fe_2_O_3_] and maghemite [γ-Fe_2_O_3_])^10^. Walker et al. show most As in the Giant Mine tailings is hosted in partly oxidized roaster calcine^5^. Therefore, in addition to sulfide oxidation, As can be mobilized by the dissolution of roaster calcine and via reductive dissolution in the saturated zone. Dissimilatory Fe(III) reduction is catalyzed by obligately anaerobic Fe(III)-reducing microorganisms (IRM), such as *Geobacter* and *Shewanella*^11^. However, many chemolithotrophic IOM (*e.g*., Fe(II)-oxidizing *Acidithiobacilli*) are facultative anaerobes capable of Fe(III) reduction^12,13^. Fe(III) reduction in acidophilic heterotrophic species has been described^14^. Importantly, obligately anaerobic heterotrophic SO_4_^2^-reducing bacteria (SRB) are known to catalyze Fe(III) reduction, supported by organic matter^15^. Co-occurrence of SRB with IRM has been reported in the past in mining environments^16–18^, and their abundance often increases with depth^19,20^. A negative effect of indirect reduction of Fe(III)-bearing minerals by SRB on the abundance of IRM have been previously observed in alkaline mine tailings^21^.

Understanding the role of bacterial and archaeal communities (BACs) in mediating the release and attenuation of As in the tailings impoundment is an important component of evaluating proposed remediation strategies. This study for the first time describes the BACs throughout the vertical tailings profile of the NW-TCA at the Giant Mine, complementing hydrological, mineralogical, and geochemical studies.

## Results

### Tailings mineralogy and geochemistry

**Figure 1:**
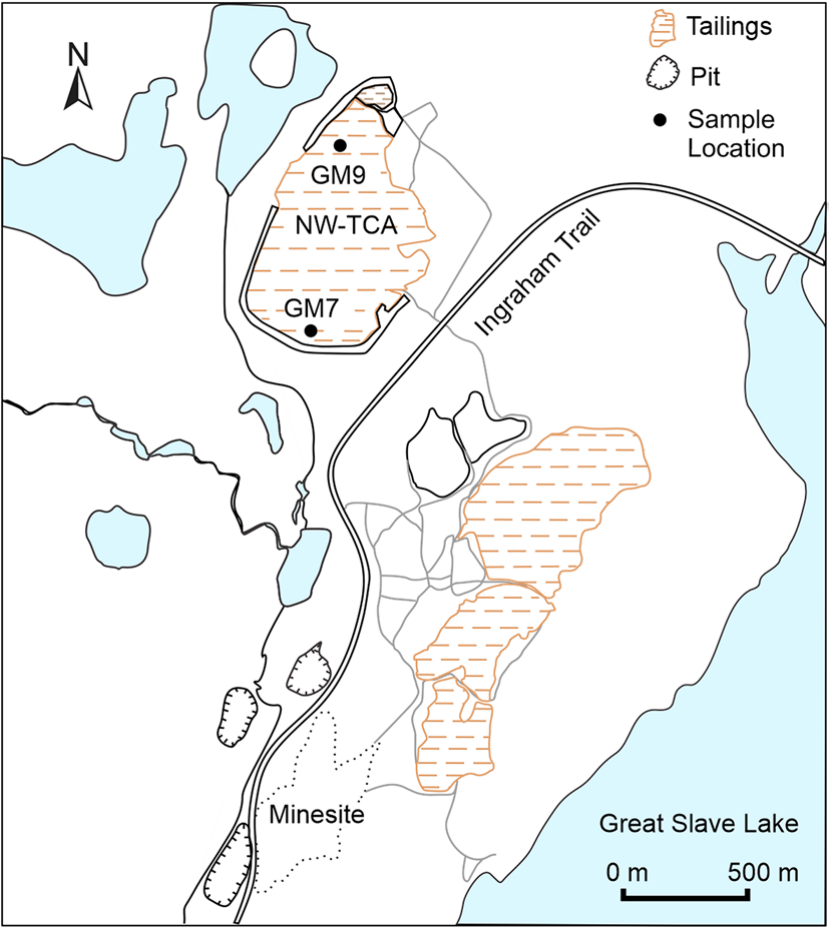
Site map of tailings impoundments at the Giant Mine showing the locations of sampling sites (GM7 and GM9) within the Northwest Tailings Containment Area (NW-TCA) for characterization.

The tailings depth ranges from 22 m in the interior of the NW-TCA to 9 m toward the peripheries. In August 2018, the water table was at depths of 10.9 and 3.2 m at the GM7 and GM9 sampling locations, respectively (Fig. 1). Tailings samples primarily comprise Si, Ca, Fe, Mg, and Al, without any systematic variations versus depth. The total inorganic C content ranges from 1.96 to 2.75 wt% and the mean sulfide-S content is 0.4 ± 0.2 wt% (mean ± s.d., n = 19), ranging from 0.16 to 1.26 wt%. The mean concentration of As within the solid phase is 2750 ppm. Arsenic-bearing hosts were identified as arsenopyrite, arsenical pyrite, and arsenic incorporated within roaster calcine (composed of maghemite and hematite), determined by optical microscopy and scanning electron microscopy and energy dispersive X-ray spectroscopy (SEM–EDS) and corroborated by a previous study ^2^.

Major mineral phases (> 10 wt%) identified by the Relative Intensity Ratio (RIR) analysis of X-ray diffraction (XRD) patterns (Fig. S1) are quartz (SiO_2_), muscovite (K_0.74_Na_0.17_Ca_0.01_Mg_0.02_Ti_0.02_Fe_0.03_A_l2.83_Si_3.1_O_10_(OH)_2_), dolomite (CaMg(CO_3_)_2_), and chlorite (identified primarily as Fe-bearing clinochlore; Mg_2.8_Fe_1.75_Al_2.7_Si_2.65_O_10_OH_8_). Minor mineral phases (< 10 wt.%) identified in all measured patterns are calcite (CaCO_3_) and rutile (TiO_2_). Feldspar plagioclase, albite Na(AlSi_3_O_8_), is identified in all measured samples with the exception of 9.7 m depth at GM7. Trace phases (< 5 wt%) are pyrite and gypsum. Iron is under-represented in the mineral assemblage identified with XRD relative to X-ray fluorescence (XRF) analysis. The only Fe-bearing mineral fit to the pattern is clinochlore-1MIIb ((Fe^2+^,Mg)_5_Al(AlSi_3_O_10_)(OH)_8_). A component of Fe-oxides were derived from roasting (9 wt%; based on XRF^2^).

There are no major trends in mineral assemblage observed in depth or location. A minor trend is observed with the percent composition of pyrite increasing with depth, markedly between 6.3 and 7.8 m at GM7 and 5.2 and 6.4 m at GM9. As RIR is a semi-quantitative technique, values of wt% pyrite should be considered relative rather than absolute values. The mineral phase pyrite was identified in all GM7 samples with the exception of 0.6 and 5.0 m, but on average 0.2 wt% between 0 and 6.3 m and 2.8 wt% between 7.8 and 12 m. As this transition is sharp and at a depth observed to be below where active oxidation of sulfide minerals was determined to have occurred, this change in composition can be attributed to differences in ore processing. Similar trends were observed in GM9 samples. The mineral composition indicates a very well buffered system in regard to acid generation, where 50–78% of the mineral assemblage is composed of either Ca–Mg carbonate phases or aluminosilicates, whereas the percent weight composition of Fe–S minerals is 0.4 ± 0.2 wt%. This is reflected in the porewater geochemistry in that the system is of neutral pH and elevated in Ca, Mg, and HCO_3_^−^ as neutralization products.

**Figure 2:**
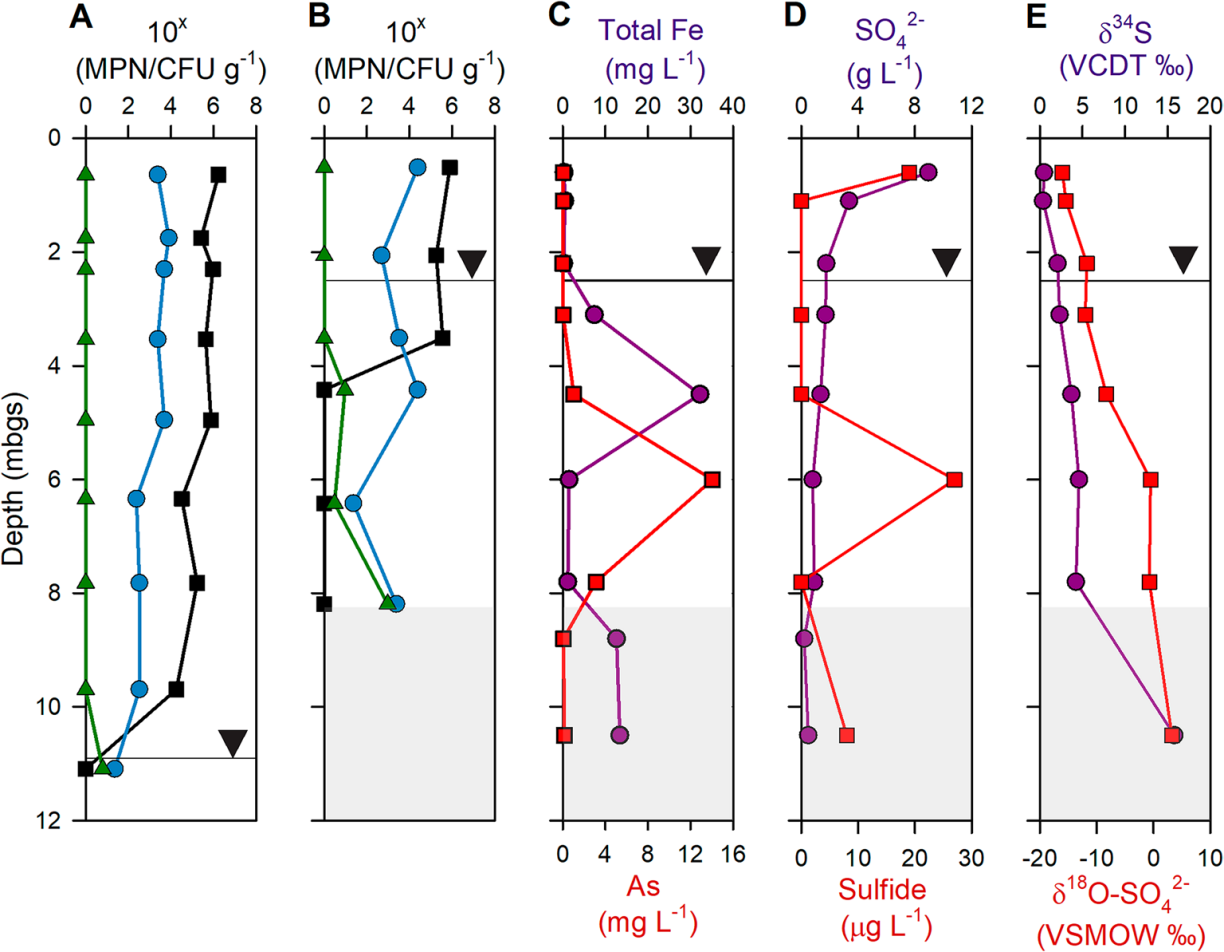
Vertical profiles for microbial counts of (green triangle) SO_4_^2−^-reducers, (blue circle) S-oxidizers, and (black square) heterotrophs in tailings collected from the NW-TCA at the Giant Mine, determined by the most probable number (MPN) technique at (**A**) GM7 and (**B**) GM9. Vertical profiles for (**C**) total Fe (purple) and As (red) concentrations, (**D**) sulfate (purple) and S^2−^ (red) concentrations, and (**E**) δ^34^S (purple) and δ^18^O-SO_4_ (red) isotopes, all from GM9 collected in June 2022. The inverted triangle represents the water table at each location and the area filled with light grey indicates natural sediments. *CFU* colony-forming units, *VCDT* Vienna-Canyon Diablo Troilite standard, *VSMOW* Vienna Standard Mean Ocean Water 2 standard.

Concentrations of dissolved Fe, SO_4_^2−^_,_ S^2−^, δ^34^S-SO_4_^2−^, and δ^18^O-SO_4_^2−^ are presented in Fig. 2. Selected additional geochemical parameters of tailings pore-water samples are summarized in Table S1. The tailings pore water has a pH of 7.1–8.6 (mean 7.6). The redox potential and alkalinity range from 109 to 426 mV and 28–376 mg L^−1^ (as CaCO_3_), respectively, with neither parameter showing a systematic variation with depth. Concentrations of dissolved metal(loid)s vary significantly in the pore-water samples, with standard deviations of 78–306%. Although the mean concentrations of most dissolved metal(loid)s are similar between the vadose and saturated zones, some discrepancies are evident (Table S2). For example, the maximum dissolved Fe concentration (71.6 mg L^−1^) occurs in the vadose zone at GM9 (2.1 m depth), whereas the maximum As concentration (20.0 mg L^−1^) occurs in the saturated zone at GM7 (5.7 m depth) (Table S1). Dissolved organic carbon (DOC) ranges from < 0.01 to 36.4 mg L^−1^, with higher concentrations generally detected deeper in the tailings profile. Dissolved SO_4_^2−^ ranges from 0.002 to 11.6 g L^−1^, with the highest concentrations near the ground surface at GM9 (maximum of 11.6 g L^−1^) and slightly elevated at the ground surface at GM7 (0.3 g L^−1^) (Table S1). Stable temperature (8–11 °C) was observed in groundwater, with greater seasonal fluctuation in the near surface samples.

### Bacterial and archaeal communities

A total of 697513 raw sequence reads were obtained, with a mean of 21833 ± 5912 reads per sample, and a mean of 897 ± 563 operational taxonomic units per library (statistics summarized in Table S3). Significantly greater (t-test; P < 0.05) richness (described by Chao’s estimator for an OTU definition) is evident in GM9 samples (2277 ± 2226) than in GM7 samples (1205 ± 988). The mean Chao’s estimator is also greater (t-test; P < 0.05) in saturated zone samples (1733 ± 632) than in vadose zone samples (1311 ± 1183). The difference in *α*-diversity (Gini-Simpson index) is significant (t-test; P < 0.05) between GM9 (0.96 ± 0.01) and GM7 samples (0.89 ± 0.09) as well as between vadose zone (0.89 ± 0.09) and saturated zone (0.96 ± 0.01) samples.

**Figure 3:**
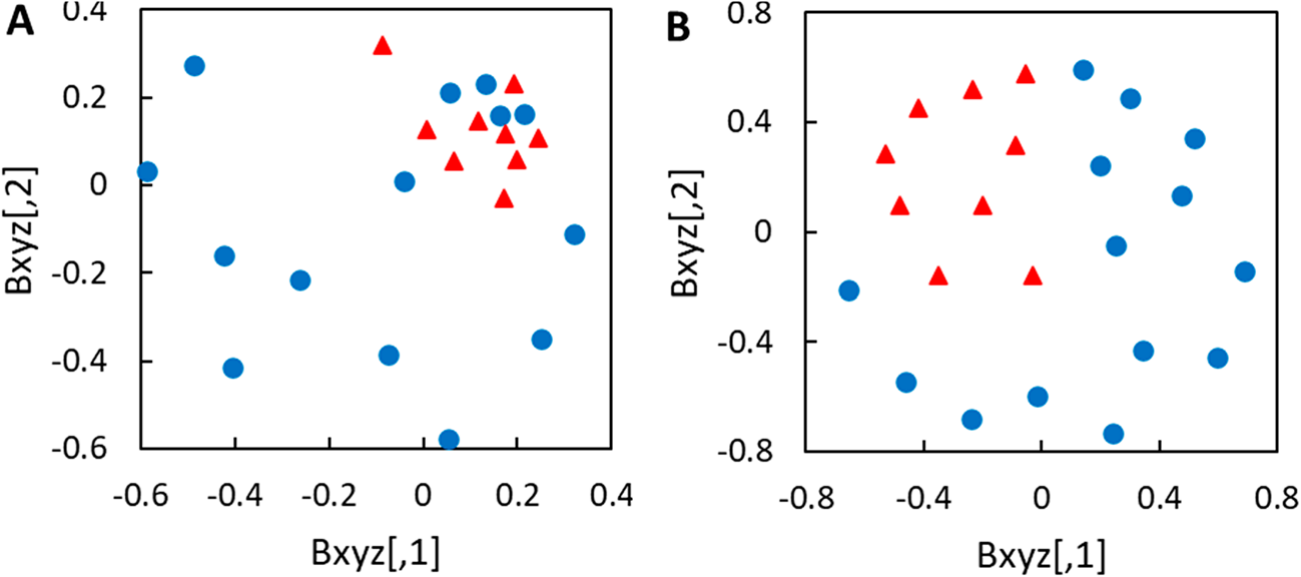
Two-dimensional non-metric multidimensional scaling (2D-NMDS) plots (stress = 0.237) of (**A**) weighted and (**B**) unweighted UniFrac used to investigate BACs in (blue circle) GM7 and (red triangle) GM9 samples. The distance between any two points represents the difference between those two communities.

Significantly greater *β*-diversity (AMOVA; P < 0.01) occurs in GM7 samples, as apparent in Fig. 3 that compares BACs in GM7 and GM9 samples in non-metric multidimensional scaling (NMDS) plots based on weighted (Fig. 3A) and unweighted (Fig. 3B) UniFrac. Significantly greater *β*-diversity (AMOVA; P < 0.05) is evident in vadose zone samples compared to saturated zone samples (not shown), reflecting the *β*-diversity difference between locations with different water table levels.

**Figure 4:**
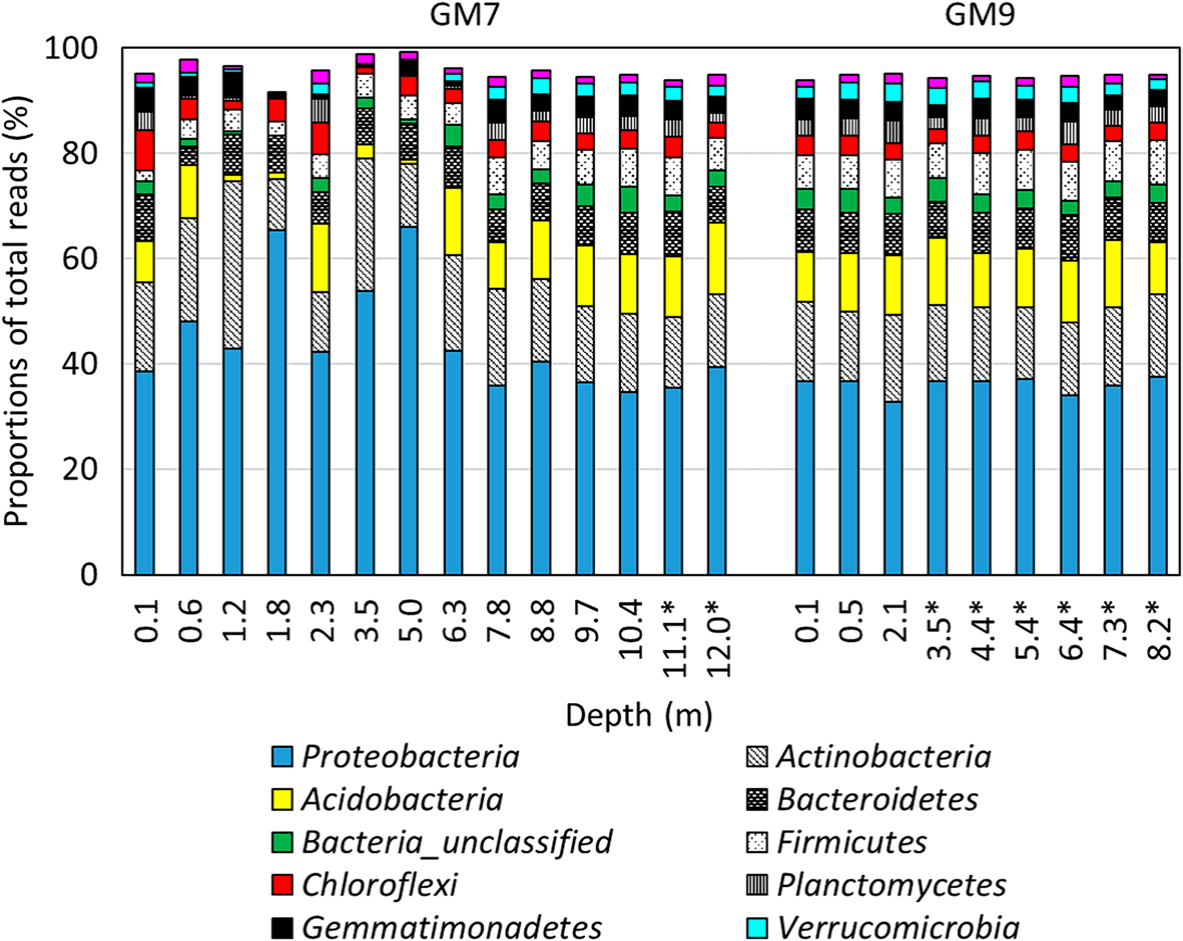
Proportions of total reads of major phyla (cut off = 1% of total reads) in NW-TCA tailings samples. Depths below the water table level are marked with an asterisk.

The phylum *Proteobacteria*, which dominates most habitats, accounts for 36.4% of the total reads in most NW-TCA tailings samples, followed by *Actinobacteria* (14.1%), *Acidobacteria* (10.5%), *Bacteroidetes* (6.6%), *Firmicutes* (5.6%), *Chloroflexi* (4.1%), *Planctomycetes* (3.7%), *Gemmatimonadetes* (3.0%), *Verrucomicrobia* (2.3%), and *Cyanobacteria* (1.4%) (Fig. 4). The phyla *Proteobacteria, Firmicutes*, and *Actinobacteria* contain species known to catalyze the dissimilatory redox reactions of Fe and/or S (such as *Acidithiobacillus*). The other detected phyla are widely distributed across a variety of geochemical settings. Mean relative abundances of archaea and unclassified bacteria respectively account for 1.2 and 6.3% of total amplicons.

Bacterial genera identified as most abundant in the NW-TCA tailings samples (Table S4) use a plethora of different metabolic strategies in a variety of habitats. A large proportion are plant symbionts (e.g., *Rhizobiales* and *Xanthobacteraceae*, accounting for 1.0% and 1.3% of total reads, respectively) or human and animal pathogens (e.g., *Enterobacteriacaea* at 2.4%).

### Fe- and S-metabolizing genera

**Figure 5:**
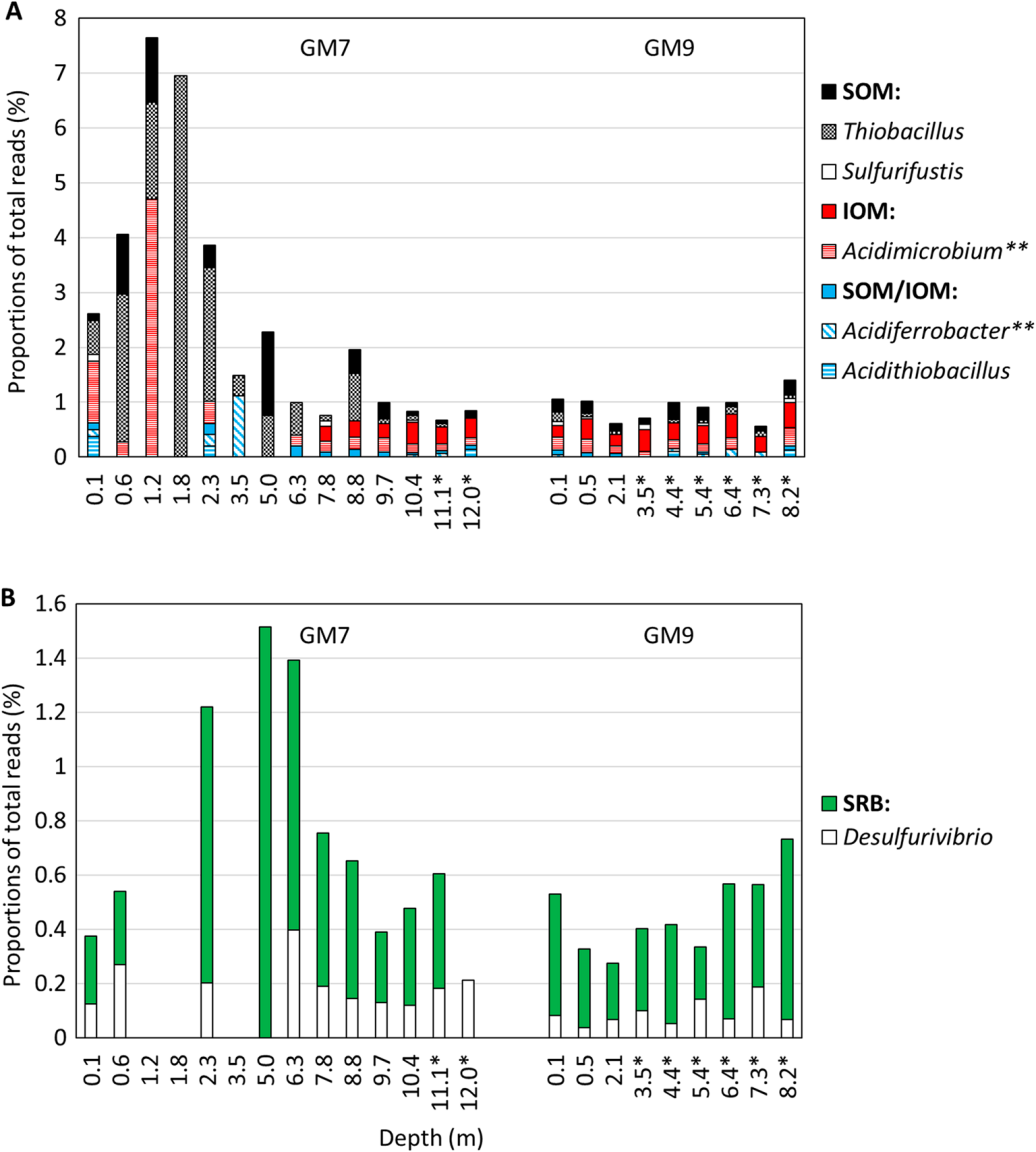
Proportions of total reads of (**A**) S- and/or Fe(II)-oxidizing and (**B**) SO_4_^2−^- and/or S-reducing bacteria and archaea (on the genus level) in NW-TCA tailings samples. SOM = S-oxidizing microorganisms (that do not oxidize Fe(II)); IOM = Fe(II)-oxidizing microorganisms (that do not oxidize S); SOM/IOM = S- and Fe(II)-oxidizing microorganisms; SRB = SO_4_^2−^- and/or S-reducing bacteria. Depths below the water table level are marked with an asterisk. Higher taxa are marked with two asterisks.

Low relative abundances of genera containing species known to metabolize Fe and/or S are present in the NW-TCA tailings samples (Fig. 5; Table S5). The neutrophilic S-oxidizing *Thiobacillus* with 0.8% of total reads is the most abundant of the mineral oxidizers detected. Significantly greater (t-test; P < 0.05) mean relative abundances of mineral oxidizers are found in vadose zone samples (2.47 ± 2.24% of total reads) than in saturated zone samples (0.88 ± 0.26%). Out of the 2.47% of all mineral oxidizers in vadose zone samples, 1.58% are SOM, 0.68% are IOM, and 0.22% are chemolithotrophs oxidizing both S and Fe. The majority of the mineral-oxidizing genera are neutrophilic, accounting for 1.54% of total reads, while acidophiles account for the rest (0.93%). The results suggest low rates of sulfide oxidation in the vadose zone.

Very low to no risk of acid mine drainage (AMD) is expected at the site, due to a low residual sulfide content in the tailings and sufficient neutralizing capacity (net neutralization potential of 38 kg CaCO_3_ tonne^−1^ at GM7 and 33 kg CaCO_3_ tonne^−1^ at GM9). Elevated SO_4_^2−^, Ca^2+^, and Mg^2+^ concentrations and alkalinity (Table S1) near the ground surface at GM9 indicate ongoing oxidation of the residual sulfides and subsequent neutralization by carbonate minerals, maintaining a circumneutral pH. In the absence of reclamation steps to inhibit O_2_ gas transport, similar conditions can be expected to persist until sulfide depletion.

Reductive dissolution of Fe oxides in the saturated zone can result in the mobilization of As from the Fe(III) oxides of the roaster calcine in NW-TCA tailings. The proportions of IRM detected are low in the saturated zone samples, with *Acidibacter* and *Geobacter* accounting for 0.30 ± 0.07 and 0.24 ± 0.16% of total reads, respectively. However, most acidophilic IOM and some SOM, as well as SRB, are capable of Fe(III) reduction under anaerobic conditions. The sum of relative abundance of all Fe(III)-reducing genera detected in saturated samples is 1.37 ± 0.34% of total amplicons. Iron and As concentrations in the tailings pore water (Table S1) increase with depth (maxima of 72 and 20 mg L^−1^, respectively), possibly due to the reduction of Fe-oxidized phases. At the base of the impoundment, the dissolved As concentration decreases to 0.4 mg L^−1^, possibly due to microbially mediated SO_4_^2−^ reduction and precipitation of As-sulfide phases.

### Numbers of culturable microorganisms

Neutrophilic SOM, SRB, and heterotrophs were enumerated by the most probable number (MPN) technique (Fig. 2). Culturable numbers of obligately anerobic SRB and aerobic heterotrophs are significantly affected by the elevation of the water table. Viable anaerobic SRB do not occur above the water table at either location, whereas aerobic heterotrophs are not detected in samples from the saturated zone. Mean values of culturable heterotrophs in samples collected from above the water table account for 5.4 × 10^5^ and 4.8 × 10^5^ CFU g^−1^ at GM7 and GM9, respectively. SOM are often facultatively anaerobic, and thus their viable numbers are not affected by the water table position. Mean SOM counts in the same samples are 2.9 × 10^3^ and 1.2 × 10^4^ MPN g^−1^, respectively. At GM9, the mean SOM count detected below the water table (8.8 × 10^3^ MPN g^−1^) is similar to that from above the water table, but a GM7 saturated-zone sample has a lower number (23 MPN g^−1^). SRB are the least abundant of the groups studied, accounting for 6 MPN g^−1^ in saturated-zone GM7 samples and 3.1 × 10^2^ MPN g^−1^ in saturated-zone GM9 samples.

## Discussion

Significantly greater richness (reflecting the abundance of OTUs) is evident in samples from GM9 compared to GM7, the latter of which has a much lower water table. A comparison of richness between vadose zone and saturated zone samples indicates the difference between the water-table elevation at the two locations is a likely cause for this difference in richness. A greater 1-D value is observed in samples from GM9 compared to GM7, but values for both locations are close to 1, signifying high *α*-diversity. Weighted and unweighted UniFrac yield similar results, indicating the abundance of individual genera does not play a significant role in the *β*-diversity assessment. In contrast to the *α*-diversity comparison, greater *β*-diversity is observed in GM7 and unsaturated zone samples than in GM9 and saturated samples, respectively.

*Proteobacteria, Nitrospirae, Actinobacteria, Firmicutes, Acidobacteria*, and *Bacteroidetes* have been reported as dominant bacterial phyla in mine-impacted environments^8,18,20,22–27^. Findings in this study are consistent with published taxonomic data, with the generally and broadly widespread *Proteobacteria* notably dominating in the NW-TCA tailings, and archaea present at low relative abundances. Elevated numbers of *Euryarchaeota* (e.g., *Ferroplasma*) are commonly observed in mine-impacted soils, but only account for 0.06% in the low-sulfide NW-TCA tailings; rather, *Thaumarchaeota* are the most abundant archaeal phylum (1.0%). *Thaumarchaeota* (some of which are NH_3_-oxidizers that play a key role in N cycling) are among the most abundant archaea on Earth and found in most soils^28^. The photosynthesizing *Cyanobacteria*, also capable of organoheterotrophy^29^ and chemolithotrophy in the presence of light^30^, have been reported in mine wastes^8,18,20^ and mine-impacted sediments^31^*.* Cyanobacteria have been hypothesized to contribute to C, S, and/or Fe cycling^20^, but could represent contamination from the surface layers^32^.

The relative abundance of IOM and/or SOM (mean of 2.5%) in the unsaturated proportion of the NW-TCA flotation/roaster tailings is significantly lower than abundances reported in mill tailings with higher sulfide contents (commonly reaching tens of percent)^23,26,33–35^ and somewhat lower than abundances in sulfidic wastes under remediation (reaching units of percent)^8,18,20^. The neutrophilic S-oxidizer *Thiobacillus* is the most abundant mineral oxidizer, reaching only 0.8% of total reads. The counts of culturable neutrophilic SOM are also relatively low (~ 10^3^ MPN g^−1^), at two to three orders of magnitude lower than counts observed in other pH-neutral mine wastes with greater sulfide contents^7,9^. For comparison, up to 10^8^ cells g^−1^ have been reported for acidophiles in acid-generating tailings^36–39^.

An additional concern at the Giant Mine is As release from the oxidized phases, produced during the roasting process, via reductive dissolution in the saturated zone. Although low proportions of obligately anaerobic IRM (e.g., *Geobacter*) are detected, other metabolic groups (such as SRB and facultatively anaerobic IOM; Table S5) are capable of Fe(III) reduction under anaerobic conditions. These species noticeably increase the total abundance of Fe(III)-reducers in the saturated zone at the NW-TCA (to 1.4%). Therefore, a certain extent of Fe(III) bio-reduction should be expected in the saturated zone.

The Fe(III)-reducing activity of SRB can lead to As mobilization from the Fe oxides in the saturated zone^10^. At GM9, an increase in the concentration of dissolved Fe and As coincides with an increase in the abundance of viable SRB. Based on the increased concentrations of soluble Fe and As, reductive dissolution of roaster calcine waste may have occurred at this depth in the saturated zone. However, SRB also catalyze SO_4_^2−^ reduction, depending on the availability of electron acceptors SO_4_^2−^ vs. Fe(III). A slight decline in the dissolved SO_4_^2−^ concentration coincides with a sharp increase in the dissolved S^2−^ concentration. SRB preferentially reduce isotopically light SO_4_^2−^ to H_2_S during sulfate reduction, resulting in enrichment of δ^34^S-SO_4_ in the water^40^. The progressive increase in the δ^34^S-SO_4_ isotope ratio below depths > 4 m is consistent with ongoing SO_4_^2−^ reduction. The sharp decrease in the dissolved Fe concentration between 6 and 8 m suggests precipitation of secondary Fe sulfides results in lower Fe concentrations, and that the rate of SO_4_^2−^ reduction exceeds the rate of Fe reduction in this zone. A sharp decrease in dissolved As concentrations detected in the natural sediments at the bottom of the NW-TCA impoundment suggests SO_4_^2−^ reduction and As sulfide precipitation might also have occurred. Heterotrophs other than SRB utilize organic C and can affect its availability. Due to the extreme metabolic diversity among heterotrophs, assessing heterotroph abundances and the use of DOC are challenging. The counts of culturable aerobic heterotrophs (reaching ~ 10^5^ CFU g^−1^ in the unsaturated zone of the NW-TCA) are lower than in undisturbed soils (1 × 10^7^ to 3.4 × 10^7^ cells g^−1^)^41^ and circumneutral mill tailings covered with an organic cover (~ 10^8^ cells g^−1^)^18^.

Microbes play a significant role in the mobilization and sequestration of As^42–46^. Prokaryotes that can gain energy from the oxidation of As(III) and/or reduction of As(V) have been described^42^. Mine waters^43–45,47^ and mill tailings^48,49^ can harbor As(III)-oxidizing prokaryotes (such as *Thiomonas*)^43,45^. Low abundances of As-metabolizing prokaryotes^42,46^ are present in the NW-TCA, with the genera *Bosea, Ralstonia, Variovorax, Ensifer,* and *Thiomonas* accounting for ≤ 0.01% of total reads. However, several groups that include species in which As-metabolism genes are found have greater abundances (e.g., *Burkholderiaceae* at 2.6%). Therefore, microbially mediated As transformations likely occur in the NW-TCA tailings. Several chemolithotrophic bacteria (*Acidithiobacillus* spp., *Shewanella putrefaciens*) have demonstrated resistance to As^50–53^, which may increase As mobilization from mine wastes.

## Conclusion

Oxidized and reduced As-bearing phases were co-disposed in the mixture of flotation tailings and roaster wastes stored at the NW-TCA. Concerns therefore include As mobilization via reductive dissolution of Fe(III) compounds in the roaster calcines in saturated tailings as well as the release of As through sulfide oxidation in the vadose zone. Furthermore, the direct dissolution of ATRW and discharge of As-containing drainage pumped from the underground workings likely contribute to the As concentrations in the pore water of the NW-TCA.

Low populations of mineral-oxidizing prokaryotes are present in the tailings, indicating low rates of ongoing sulfide oxidation in the vadose zone of the tailings impoundment. These findings are consistent with the results of geochemical analyses that show the tailings pore water has a circumneutral pH but elevated concentrations of dissolved metal(loid)s. However, most of the As in the tailings solids is associated with Fe oxides formed during roasting, with the reductive dissolution of these phases in the saturated zone representing an environmental concern. The results of this study confirm the presence of both heterotrophic and autotrophic species capable of Fe(III) reduction (although in relatively low numbers).

Due to the high As content in the NW-TCA, the application of a remediation strategy that would prevent the release and transport of As from both oxidized and reduced sulfide phases in the tailings is crucial. However, remediation efforts are complicated by the complexity of the microbiome and the extreme metabolic diversity of the prokaryotes that catalyze Fe(III) reduction. Many factors (particularly DOC, SO_4_^2−^, and Fe(III) concentrations) should be considered when developing a suitable remediation strategy for the tailings at the NW-TCA. The results of this investigation demonstrate the need to consider the impacts of triggering microbial processes in either redox direction that would result in As release from the tailings.

## Materials and methods

### Sampling

In July/August 2018, core samples were collected at two locations (GM7 and GM9; Fig. 1) in the NW-TCA to depths of 12 and 8.2 m, respectively, using a method described by Starr and Ingleton^54^. The core samples were cut into 20-cm long sections, which were capped prior to subsampling for microbiological analyses (Table 1). Core subsamples for enumerations of culturable microorganisms were stored at 4 °C until processing in the laboratory within one week of collection. Core subsamples for DNA extractions (and subsequent 16S rRNA amplicon sequencing) were stored at − 20 °C.

**Table 1:** Summary of environmental samples collected from two locations (GM7, GM9) in the Northwest Tailings Containment Area at the Giant Mine. Microbial diversity was analyzed using high-throughput amplicon sequencing of 16S rRNA genes, and active groups of microorganisms were enumerated using the most probable number (MPN) technique (samples analyzed by MPN are in bold). Depths below the water table level are marked with an asterisk.

Location	Depth (m)													
GM7	0.10	**0.64**	1.20	**1.75**	**2.30**	**3.53**	**4.95**	**6.34**	**7.82**	8.76	**9.69**	10.40	11.09*	12.00*
GM9	0.10	**0.51**	**2.06**	3.51*	4.42*	5.42*	6.42*	7.31*	8.18*	–	–	–	–	–

### Tailings solid analyses

Total C and total S contents were analyzed in the tailings solid samples in triplicate by combustion with an induction furnace (ELTRA CS800) followed by analysis of gaseous combustion products (ELTRA CS-2000). A calibration curve was generated with LECO C/S standards run in quintuplet with a calibration check performed every 12th sample. The elemental composition of the tailings solid samples was determined by XRF (Panalytical Minipal 4 desktop ED-XRF). Six representative samples were analyzed for total rock analysis at three commercial laboratories with mean values used for calibration. Samples were mixed with a cellulose based binding agent (12 wt%), homogenized in a planetary ball mill to a particle size < 80 µm, then measured as a pressed pellet. Mineral composition was analyzed by powder XRD at the CMCF-BM beamline at the Canadian Light Source (Saskatoon, Canada). The incident energy was 18 keV with a wavelength of 0.6888 angstroms. Prior to analysis, samples were finely ground with a mortar and pestle and packed into polyamine tubing. Raw diffraction rings were calibrated, background corrected, and integrated to peak patterns using GSAS II^55^. Mineral phase identification and RIR analysis was performed using the JADE and SIEVE + software packages with access to the PDF-4 mineralogical database (ICDD/MDI).

### Pore-water and groundwater analyses

Pore-water and groundwater samples were collected in the field via suction lysimeters and drive-point piezometers as well as through the immiscible liquid displacement extraction method^56^. Pore-water pH and redox potential (relative to standard hydrogen electrode) were determined immediately after extraction using a combination pH electrode (Orion 815600 Ross Combination pH Probe) and a platinum redox electrode (Orion 9678B NWD Sure-Flow Combination redox electrode), respectively, coupled to an Orion 3 Star pH/mV meter. Alkalinity titrations were performed in the field with methyl red/bromocresol green indicator and sulfuric acid using a digital titrator (Hach Chemical Company). Dissolved S^2−^ and Fe^2+^ measurements were conducted immediately after sample collection with a Hach spectrophotometer by the methylene blue method (Hach Method 8131) and 1,10-phenanthroline method (Hach Method 8146), respectively. Samples for determination of dissolved cation and anion concentrations were filtered (0.45 µm; PVDF) and preserved (HNO_3_; pH < 2.0 for cations) in the field and stored at 4 °C until analysis. Inductively coupled plasma-optical emission spectrometry (ICP-OES ICAP 6000, Thermo Scientific)^57^ and inductively coupled plasma-mass spectrometry (ICP-MS X Series II, Thermo Scientific)^58^ were used to determine cation concentrations; ion chromatography (Dionex IC-CO_3_ system)^59^ was used to measure anion concentrations. Filtered (0.45 μm) aqueous samples (preserved in H_2_SO_4_; pH ~ 2.0) were analyzed for DOC using wet oxidation with heated Na_2_S_2_O_8_ (Aurora 1030W TOC Analyzer, OI Analytical). Inorganic carbon was removed from the sample by the instrument with the addition of 5% H_3_PO_4_. Measurement of δ^34^S and δ^18^O-SO_4_^2−^ isotopes was conducted by the Environmental Isotope Laboratory, Waterloo, ON using a BaSO_4_ precipitation method (Isochrom CFRIMS, Micromass, UK; Isoprime CFIMS, GV Instruments).

### Microbiological analyses

Extraction of total genomic DNA from duplicate tailings samples using the DNeasy PowerSoil Kit (Qiagen Inc., Germany), amplification of the V4 region of 16S rRNA genes (by Metagenom Bio Inc., Toronto, Canada), and subsequent Illumina MiSeq sequencing (also by Metagenom Bio Inc.) were completed using the methods described by Pakostova et al.^10^. Three samples were sequenced in analytical triplicate. Sequence data were analyzed using mothur software v.1.39.5, updated: 3/20/2017^60^, and the mothur MiSeq Standard Operating Procedure^61^; https://www.mothur.org/wiki/MiSeq_SOP) from 12/10/2018. Two out of 46 samples in total contained fewer than 10,000 sequences and were removed prior to duplicate (or triplicate) merging. A detailed description of sequence data processing can be found elsewhere^8,18^.

The MPN technique^62,63^ was used to enumerate different metabolic groups of culturable microorganisms. SOM, SRB, and heterotrophs were enumerated as described by Pakostova et al.^18^. In short, aerobic SOM were cultivated in a liquid basal-salt medium (pH ~ 7.0) containing 5 g L^−1^ Na_2_S_2_O_3_·5H_2_O as the substrate; anaerobic SRB were cultivated in a modified Postgate C medium (pH ~ 7.5) containing 2.92 g L^−1^ Na lactate (60%) and 1.28 g L^−1^ Na acetate; and aerobic (and facultatively anaerobic) heterotrophs were cultivated on R2A agar (Sigma Aldrich, USA; pH ~ 7.2). All cultivations were conducted at room temperature (~ 23 °C), without agitation. A sample collected at 3.52 m depth at GM9, immediately below the water table position (~ 3.2 m), was disregarded for the MPN comparisons, considering fluctuations in the water table and the presence of the tension-saturated zone above the water table.

### Supplementary Information

Supplementary Information.

## Data Availability

Illumina sequence data used in this study have been deposited in the European Nucleotide Archive (ENA) at EMBL-EBI under accession number PRJEB57552 (https://www.ebi.ac.uk/ena/browser/view/PRJEB57552).

## Abbreviations

AMD: Acid mine drainage
AMOVA: Analysis of molecular variance
BAC: Bacterial and archaeal community
DOC: Dissolved organic carbon
IOM: Fe(II)-oxidizing microorganisms
IRM: Fe(III)-reducing microorganisms
MPN: Most probable number
NMDS: Non-metric multidimensional scaling
NW-TCA: Northwest Tailings Containment Area
OTU: Operational taxonomic unit
SOM: S-oxidizing microorganisms
SRB: SO_4_^2^^−^- and/or S-reducing bacteria
