# Supplementary material for: Microbial processes with the potential to mobilize As from a circumneutral-pH mixture of flotation and roaster tailings
Source: Sci Rep. 2023 Dec 27;13:23048. doi: 10.1038/s41598-023-50435-3 (PMC10754864; doi:10.1038/s41598-023-50435-3)
Supplement: Supplementary file 1 — Supplementary Information. [file 41598_2023_50435_MOESM1_ESM.pdf]

Supplemental materials for

Microbial processes with the potential to mobilize As from a circumneutral-pH mixture of flotation and roaster tailings

Eva Pakostova[a,b]*, David M. Hilger[a], David W. Blowes[a], Carol J. Ptacek[a]

[a]*Department of Earth and Environmental Sciences, University of Waterloo, Waterloo, Canada.*

[b]*Centre for Manufacturing and Materials, Coventry University, Coventry, UK.*

*Corresponding author: Eva Pakostova: 150560@mail.muni.cz, +1 519-888-4567, ext. 36431

David M. Hilger: david.hilger@uwaterloo.ca, +1 519-888-4567, ext. 37232

David W. Blowes: blowes@uwaterloo.ca, +1 519-888-4567, ext. 84878

Carol J. Ptacek: ptacek@uwaterloo.ca, +1 519-888-4567, ext. 32230

Table S1. Selected available physicochemical characteristics of tailings samples (determined in pore water) collected from the Northwest Tailings Containment Area at the Giant Mine at depths close to samples subjected to microbiological analyses. Depths marked with asterisks are below the water table level. Data are not available for depths greater than 5.7 m at GM7 due to the lack of success in acquiring pore water from the core samples. Legend: DOC = dissolved organic C.

Location	Depth (m)	pH	Eh (mV)	Alkalinity (mg L^{-1} as $CaCO_3$)	SO_4^{2-} (mg L^{-1})	DOC (mg L^{-1})	Fe (mg L^{-1})	As (mg L^{-1})	Ca (mg L^{-1})	Mg (mg L^{-1})
GM7	0.1	7.75	378.7	93.2	329	<0.01	0.04	0.43	129.0	33.5
	0.6	7.56	366.3	190.8	2.9	<0.01	0.01	0.24	200.8	43.3
	1.1	7.58	420.6	280.5	2	<0.01	0.14	0.63	593.9	184.1
	1.8	n.a.	n.a.	n.a.	4.6	<0.01	0.11	0.13	587.6	186.2
	2.5	7.19	381.5	104.2	5.2	<0.01	0.1	0.67	582.1	173.6
	3.8	7.45	401.2	n.a.	11.4	36.39	0.03	0.33	654.1	232.6
	4.7	n.a.	n.a.	n.a.	13.4	29.51	0.27	0.10	564.1	192.1
	5.7	7.65	381.2	347.8	2.4	17.62	0.06	20.02	542.0	222.4
GM9	0.1	7.64	425.6	158.8	11,606	n.a.	0.1	2.66	484.0	2,735
	0.5	7.42	402.3	n.a.	5,510	n.a.	0.12	0.87	591.5	986.6
	2.1	7.14	108.9	375.5	1,996	7.52	71.6	0.03	551.9	314.5
	3.1(*)	7.18	220	344.8	1,839	8.19	3.81	3.17	668.9	231.1
	4.5*	7.94	406	351.4	1,010	7.33	0.18	10.03	230.3	180.3
	6*	8.56	329.5	40.1	714	33.09	1.88	14.59	272.1	11.4
	7.5*	7.92	391.1	28.1	841	30.98	1.26	4.05	244.1	43.2
	10.5*	7.06	201.6	95.8	571	27.07	13.9	0.41	242.5	103.6

Table S2. Aqueous concentrations of transition metals and metalloids in tailings pore-water samples from the Northwest Tailings Containment Area.

Metal(loid)	Concentration (mg L^{-1}) (mean ± s.d.)	
	Vadose zone (n = 11)	Saturated zone (n = 5)
Al	0.06 ± 0.03	0.21 ± 0.17
As	2.37 ± 5.90	6.45 ± 5.75
Co	0.48 ± 0.60	0.20 ± 0.15
Cu	0.02 ± 0.01	0.01 ±0.01
Fe	6.60 ± 21.8	4.20 ± 5.59
Ni	0.48 ± 0.85	0.26 ± 0.50
Mn	2.54 ± 3.04	2.09 ± 3.02
Sb	3.79 ± 5.10	2.44 ± 2.06
Zn	0.61 ± 0.62	0.17 ± 0.31

Table S3. Total numbers of raw and effective sequences, OTU (operational taxonomic unit) counts, Good's coverage (calculated for an OTU definition of 0.03), Chao's species richness, and α-diversity Simpson index in the Giant Mine samples. Legend: Depths below the water table level are marked with an asterisk.

Location	Depth (m)	Total raw sequences	Effective sequences for analysis	OTU counts	Good's coverage	Chao's estimator	Gini-Simpson index (1-D)
GM7	0.10	21,736	18,205	621	0.976	858	0.978
	0.64	43,744	33,039	233	0.993	315	0.893
	1.20	36,673	32,174	102	0.996	135	0.830
	1.75	22,777	12,695	60	0.997	92	0.763
	2.30	26,342	18,543	400	0.986	616	0.956
	3.53	33,115	25,405	170	0.991	536	0.936
	4.95	18,870	9,500	132	0.993	327	0.652
	6.34	34,565	25,159	338	0.988	503	0.839
	7.82	31,154	21,784	790	0.966	1,111	0.841
	8.76	38,323	26,831	970	0.966	1,335	0.915
	9.69	33,417	23,509	1,561	0.918	2,591	0.971
	10.40	29,439	19,214	1,776	0.896	3,251	0.960
	11.09*	34,253	24,070	1,195	0.949	1,826	0.946
	12.00*	47,277	33,170	870	0.968	1,253	0.960
GM9	0.10	29,605	20,812	1,725	0.906	2,920	0.962
	0.51	36,168	24,763	1,639	0.894	3,483	0.939
	2.06	26,258	19,010	1,180	0.959	1,599	0.971
	3.51*	30,107	22,718	751	0.978	978	0.948
	4.42*	23,688	17,808	1,478	0.930	2,297	0.962
	5.42*	25,124	18,928	1,463	0.911	2,815	0.973

	6.42*	22,977	16,866	1,194	0.959	1,589	0.964
	7.31*	26,941	19,637	862	0.974	1,096	0.971
	8.18*	24,960	18,328	1,116	0.937	2,007	0.945

Table S4. Mean proportions of total reads of major bacterial genera (mean relative abundance >0.5% of total amplicons) and sum of minor genera (<0.5%) determined in Giant Mine tailings samples. Higher taxa that could not be identified on the genus level are marked with asterisks.

Genus	Mean % of total reads
Sum of minor genera	53.71
*Bacteria**	6.27
Subgroup_6_ge	4.30
*Betaproteobacteriales**	3.10
*Gammaproteobacteria**	2.78
*Burkholderiaceae**	2.56
*Enterobacteriaceae**	2.39
*Chitinophagaceae**	1.33
*Alphaproteobacteria**	1.33
*Actinobacteria**	1.32
*Xanthobacteraceae**	1.30
*Lachnospiraceae**	1.23
*Subgroup_6**	1.21
*Sphingomonadaceae**	1.16
Chloroplast_ge	1.05
uncultured_ge	1.03
*Rhizobiales**	1.00
67-14_ge	0.92
*Proteobacteria**	0.90
Omnitrophicaeota_ge	0.87
uncultured	0.85
*Actinobacteria**	0.75
Pedosphaeraceae_ge	0.73

Haliangium	0.69
Gemmatimonas	0.67
*Gaiellales**	0.67
*Deltaproteobacteria**	0.67
*Myxococcales**	0.67
Nitrososphaeraceae_ge	0.66
RB41	0.62
*Xanthomonadaceae**	0.57
WD2101_soil_group_ge	0.56
uncultured	0.56
*Gemmatimonadaceae**	0.54
KD4-96_ge	0.53
*Micrococcales**	0.51

Table S5. Summary of weighted relative abundances (considering different numbers of genera in the samples) of microorganisms that oxidize both S and Fe(II) (SOM/IOM), oxidize Fe(II) (but not S; IOM), oxidize S (but not Fe(II); SOM), or reduce Fe(III) (IRM) or SO_4^{2-} (and/or S; SRB) detected in tailings samples from the Northwest Tailings Containment Area at the Giant Mine. Higher taxa that could not be identified on the genus level are marked with asterisks. '+' indicates at least one species of the genus has been reported to catalyze the given dissimilatory reaction. EA=extremely acidophilic, MA=moderately acidophilic, N=neutrophilic, A=alkaliphilic.

Genus	Mean % of total reads	Mean % of total reads (vadose zone)	Mean % of total reads (saturated zone)	pH preference	S oxidation	Fe(II) oxidation	SO_4^{2-} (and S) reduction	Fe(III) reduction[1]
**SOM/IOM**								
*Acidiferrobacteraceae**	0.05	0.06	0.02	EA	+	+	-	+
Acidiferrobacter	0.04	0.04	0.03	EA	+	+	-	+
Acidithiobacillus	0.04	0.04	0.05	EA	+	+	-	+
Alicyclobacillus	0.03	0.04	0.02	EA & MA	+	+	-	+
Sulfobacillus	0.02	0.03	0.01	EA	+	+	-	+
**IOM**								
*Acidimicrobiia**	0.41	0.54	0.15	EA	-	+	-	+
*Gallionellaceae**	0.08	0.05	0.14	N (& MA)	-	+	-	-
Sideroxydans	0.07	0.04	0.11	N (& MA)	+	+	-	-
Gallionella	0.04	0.03	0.07	N (& MA)	-	+	-	-
Ferrovibrio	0.01	0.01	0.01	N	-	+	-	-
Ferritrophicum	0.01	0.01	0.02	N (& MA)	-	+	-	-
Leptospirillum	<00.01	<0.01	0.01	EA	-	+	-	
**SOM**								

Thiobacillus	0.79	1.18	0.06	N	+	-	-	-
Sulfuriferula	0.08	0.11	0.01	MA (& N)	+	-	-	-
Sulfuritalea	0.06	0.06	0.06	N	+	-	-	-
Sulfuricella	0.05	0.07	0.01	N	+	-	-	-
Acidicaldus	0.04	0.03	0.05	EA	+	-	-	+
*Sulfuricellaceae**	0.04	0.05	<0.01	N	+	-	-	-
Sulfurifustis	0.03	0.03	0.03	N	+	-	-	-
Acidiphilium	0.02	0.02	0.01	EA	+	-	-	+
Thiomonas	0.01	<0.01	0.01	MA	+	-	-	-
Sulfurirhabdus	0.01	0.02	<0.01	N	+	-	-	-
Sulfurimonas	<0.01	0.01	<0.01	N	+	-	-	-
Sulfuricurvum	<0.01	<0.01	<0.01	N	+	-	-	-
**IRM**								
Acidibacter	0.30	0.30	0.30	A	-	-	-	+
Geobacter	0.14	0.09	0.24	N	-	-	-	+
**SRM**								
Desulfurivibrio	0.12	0.12	0.13	A	-	-	+	+
Desulfosporosinus	0.08	0.06	0.10	N & MA	-	-	+	+
Desulfovibrio	0.04	0.04	0.03	N & MA	-	-	+	+
*Desulfobulbaceae**	0.04	0.04	0.04	N	-	-	+	+
Desulfobacca	0.04	0.03	0.05	N	-	-	+	+
Desulfatiglans	0.04	0.06	0.01	N	-	-	+	+
*Desulfobacteraceae**	0.03	0.04	0.02	N & MA	-	-	+	+
*Desulfuromonadales**	0.02	0.01	0.04	N	-	-	+	+
Dethiobacter	0.02	0.02	0.02	A	-	-	+	+
Desulfobulbus	0.02	0.03	0.02	N	-	-	+	+
Desulfuromonas	0.02	0.02	0.01	N	-	-	+	+
Desulfobacter	0.01	0.01	<0.01	N & MA	-	-	+	+
Desulfococcus	0.01	0.01	<0.01	MA & N	-	-	+	+
Desulfuromusa	0.01	0.01	<0.01	N	-	-	only sulfur	+
Desulfitobacterium	0.01	0.02	<0.01	N & MA	-	-	+	+
Desulfotomaculum	0.01	0.01	<0.01	N & A	-	-	+	+

Desulfovirga	<0.01	< 0.01	0.01	N	-	-	+	+
Desulfitibacter	<0.01	< 0.01	<0.01	N & A	-	-	+	+
*Desulfarculaceae**	<0.01	< 0.01	<0.01	N	-	-	+	+
Desulfatirhabdium	<0.01	< 0.01	<0.01	N	-	-	+	+
Desulfatitalea	<0.01	< 0.01	0.01	N	-	-	+	+
Desulfomonile	<0.01	< 0.01	<0.01	N & MA	-	-	+	+

[1]both direct and indirect

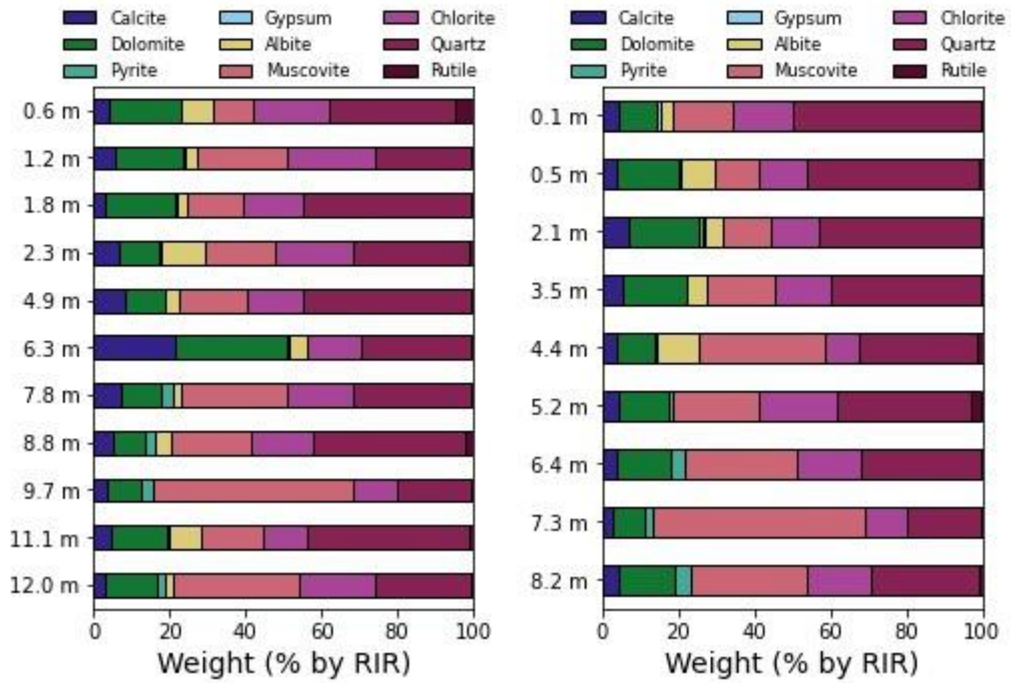

Fig. S1. Results of the Relative Intensity Ratio (RIR) analysis of X-ray diffraction (XRD) patterns of samples from GM7 (left) and GM9 (right). The XRD analysis is of 18 different patterns.
